# KIdney aNd blooD prESsure ouTcomes in Childhood Cancer Survivors: Description of Clinical Research Protocol of the KINDEST-CCS Study

**DOI:** 10.1177/20543581221130156

**Published:** 2022-10-27

**Authors:** Adree Khondker, Michael Groff, Sophia Nunes, Carolyn Sun, Natasha Jawa, Jasmine Lee, Vedran Cockovski, Yasmine Hejri-Rad, Rahul Chanchlani, Adam Fleming, Amit Garg, Nivethika Jeyakumar, Abhijat Kitchlu, Asaf Lebel, Eric McArthur, Luc Mertens, Paul Nathan, Rulan Parekh, Serina Patel, Jason Pole, Raveena Ramphal, Tal Schechter, Mariana Silva, Samuel Silver, Lillian Sung, Ron Wald, Paul Gibson, Rachel Pearl, Laura Wheaton, Peter Wong, Kirby Kim, Michael Zappitelli

**Affiliations:** 1 Child Health Evaluative Sciences, The Hospital for Sick Children, Toronto, ON, Canada; 2 Temerty Faculty of Medicine, University of Toronto, ON, Canada; 3 Department of Epidemiology and Biostatistics, Schulich School of Medicine and Dentistry, The University of Western Ontario, London, Canada; 4 Division of Nephrology, Department of Pediatrics, The Hospital for Sick Children, Toronto, ON, Canada; 5 Department of Pediatrics, McMaster Children’s Hospital, Hamilton, ON, Canada; 6 Department of Pediatric Hematology/Oncology, McMaster Children’s Hospital, Hamilton, ON, Canada; 7 Department of Medicine, London Health Sciences Centre Research Inc., London, ON, Canada; 8 Institute of Clinical Evaluative Sciences Western, London, ON, Canada; 9 Division of Nephrology, Department of Medicine, University of Toronto, ON, Canada; 10 Institute for Clinical Evaluative Sciences, Toronto, ON, Canada; 11 Division of Cardiology, The Labatt Family Heart Center, The Hospital for Sick Children, Toronto, ON, Canada; 12 Division of Hematology/Oncology, Department of Pediatrics, The Hospital for Sick Children, Toronto, ON, Canada; 13 Department of Pediatric Hematology/Oncology, Children’s Hospital of Western Ontario, London, Canada; 14 Pediatric Oncology Group of Ontario, Toronto, Canada; 15 Department of Pediatrics, Children’s Hospital of Eastern Ontario–Ottawa Children’s Treatment Centre, Canada; 16 Department of Pediatrics, Kingston Health Sciences Centre, ON, Canada; 17 Division of Nephrology, Department of Medicine, Queen’s University, Kingston, ON, Canada; 18 Unity Health Toronto, ON, Canada; 19 William Osler Health System, Brampton, ON, Canada; 20 Patient Partner, The Hospital for Sick Children, Toronto, ON, Canada

**Keywords:** cancer survivors, children, chronic kidney disease, hypertension, protocol

## Abstract

**Background:**

Approximately 30% of childhood cancer survivors (CCSs) will develop chronic kidney disease (CKD) or hypertension 15 to 20 years after treatment ends. The incidence of CKD and hypertension in the 5-year window after cancer therapy is unknown. Moreover, extent of monitoring of CCS with CKD and associated complications in current practice is underexplored. To inform the development of new and existing care guidelines for CCS, the epidemiology and monitoring of CKD and hypertension in the early period following cancer therapy warrants further investigation.

**Objective:**

To describe the design and methods of the KIdney aNd blooD prESsure ouTcomes in Childhood Cancer Survivors study, which aims to evaluate the burden of late kidney and blood pressure outcomes in the first ~10 years after cancer therapy, the extent of appropriate screening and complications monitoring for CKD and hypertension, and whether patient, disease/treatment, or system factors are associated with these outcomes.

**Design:**

Two distinct, but related studies; a prospective cohort study and a retrospective cohort study.

**Setting:**

Five Ontario pediatric oncology centers.

**Patients:**

The prospective study will involve 500 CCS at high risk for these late effects due to cancer therapy, and the retrospective study involves 5,000 CCS ≤ 18 years old treated for cancer between January 2008 and December 2020.

**Measurements:**

Chronic kidney disease is defined as Estimated glomerular filtration rate <90 mL/min/1.73 m^2^ or albumin-to-creatinine ratio ≥ 3mg/mmol. Hypertension is defined by 2017 American Academy of Pediatrics guidelines.

**Methods:**

Prospective study: we aim to investigate CKD and hypertension prevalence and the extent to which they persist at 3- and 5-year follow-up in CCS after cancer therapy. We will collect detailed biologic and clinical data, calculate CKD and hypertension prevalence, and progression at 3- and 5-years post-therapy. Retrospective study: we aim to investigate CKD and hypertension monitoring using administrative and health record data. We will also investigate the validity of CKD and hypertension administrative definitions in this population and the incidence of CKD and hypertension in the first ~10 years post-cancer therapy. We will investigate whether patient-, disease/treatment-, or system-specific factors modify these associations in both studies.

**Limitations:**

Results from the prospective study may not be generalizable to non-high-risk CCS. The retrospective study is susceptible to surveillance bias.

**Conclusions:**

Our team and knowledge translation plan is engaging patient partners, researchers, knowledge users, and policy group representatives. Our work will address international priorities to improve CCS health, provide the evidence of new disease burden and practice gaps to improve CCS guidelines, implement and test revised guidelines, plan trials to reduce CKD and hypertension, and improve long-term CCS health.

## Introduction

### Chronic Kidney Disease and Hypertension Among Childhood Cancer Survivors

Long-term complications are common in childhood cancer survivors (CCSs).^1-3^ Over 30% of adult CCS have chronic kidney conditions more than 15 years post-diagnosis and are observed to have an increased risk of chronic kidney disease (CKD) and hypertension compared with non-CCS.^4-6^ Nephrotoxic cancer therapies in children can trigger acute or chronic kidney outcomes such as acute kidney injury (AKI), CKD, and hypertension.^7-9^ Chronic kidney disease, proteinuria, and hypertension prevalence rates range from 2% to 32%, 4% to 84%, and 50%, respectively, in CCS at varying follow-up time-points,^2,10^ and there is evidence that these cardiovascular risk factors may increase morbidity, mortality, and reduce quality of life (QoL).^11-14^

Research on long-term effects of cancer therapy on kidney function in CCS has been historically limited by small sample size and suboptimal definitions for CKD and hypertension with resulting uncertainty of the onset, severity, and characteristics of these conditions in CCS.^2^,^15-17^ Furthermore, most studies on kidney or blood pressure (BP) outcomes in CCS are performed more than 5 years after cancer therapy. The first 5 years post-therapy may be a critical window to mitigate long-term progression and adverse effects of CKD and hypertension in CCS.

Current kidney and BP follow-up guidelines (eg, Children’s Oncology Group [COG]),^18^ lack specific or actionable recommendations for detecting, preventing, or treating CKD and hypertension soon after cancer therapy completion. The “Kidney Disease: Improving Global Outcomes (KDIGO)”^19,20^ and Pediatric Hypertension^21^ guidelines do provide actionable recommendations on kidney disease and BP monitoring, although not specific to CCS. To our knowledge, there have been no attempts to harmonize these guidelines to optimize practice. Moreover, the extent of CKD and hypertension monitoring being performed in CCS during the first 5 years after cancer therapy completion is unknown.

### Study Aims

To investigate CKD and hypertension epidemiology during the first 5 years after cancer therapy and identify evidence-practice gaps in contemporary screening and prevention of CKD and hypertension, and of complications from CKD and hypertension in CCS.

## Methods

### KINDEST-CCS Study Overview

**Figure 1.**
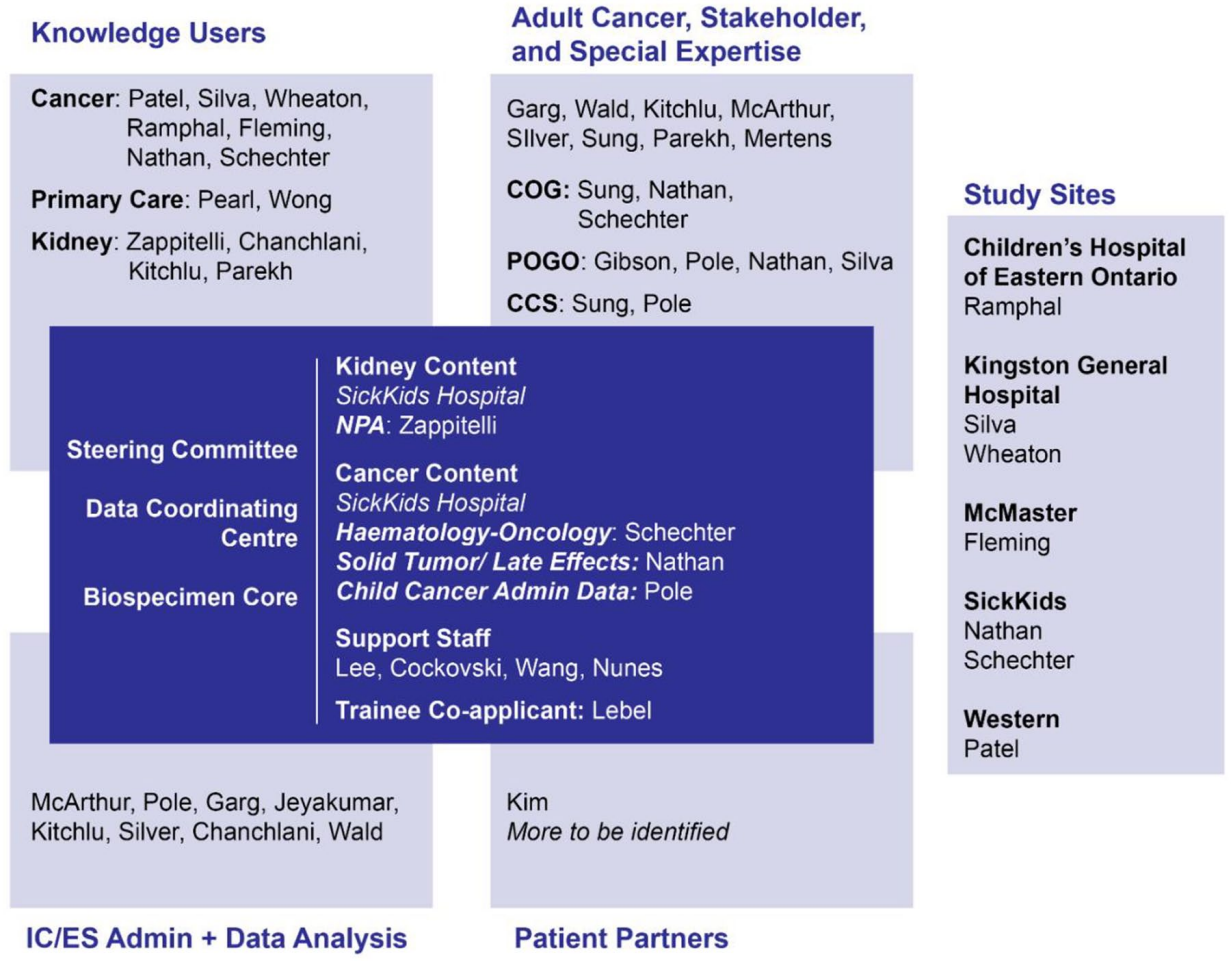
Study organizational structure of KINDEST-CCS.

We will perform 2 related multi-center studies in Ontario, Canada. This pair of studies involves a network of clinicians, researchers, biostatisticians, stakeholders, and patient partners (Figure 1 and Supplementary Table 1). Both studies will be conducted at 5 pediatric oncology sites in Ontario, Canada: Hamilton, Kingston, London, Ottawa, and Toronto.

A prospective cohort study will investigate CKD and hypertension epidemiology in CCS during the first 5 years post-cancer therapy. A retrospective cohort study will use provincial administrative healthcare data and institutional health records to highlight kidney monitoring and practice gaps in CCS and to validate administrative CKD and hypertension definitions. We will also investigate the extent to which patient, disease, treatment, or system factors are associated with or modify kidney and BP outcomes and/or monitoring practices. These studies are necessary first steps toward developing and reshaping evidence-based guidelines, for better kidney outcomes in CCS.

### Study Organization

The Hospital for Sick Children (SickKids) and the SickKids Research Institute will act as a participating site, study and data coordinating center, and the biorepository. ICES (a not-for-profit provincial research institute holding Ontario administrative health data) scientists and analysts will be integral members of the study team, receiving secure study data for linkage and analysis with administrative healthcare data.^22-24^

SickKids will provide comprehensive training on the study protocol via video-teleconference covering data collection, biospecimen collection and processing, standardized physical exams and placement of 24-hour ambulatory blood pressure monitoring (ABPM) devices. The study will be submitted to the Ontario Cancer Research Ethics Board (OCREB) for approval, prior to study start (at this time, the Team is finalizing case report forms and online database). Upon approval, we anticipate recruitment for the prospective study will begin in 2022, with a recruitment target of 500 patients.

## Prospective Study: Epidemiology of Kidney and BP Outcomes in the 5 Years After Therapy

### Prospective Study Aims

1. Evaluate CKD and hypertension prevalence at 3 and 5 years after cancer therapy and prevalence of 24-hour ABPM abnormalities at 5 years after cancer therapy, in CCS at high risk for late kidney and BP outcomes.
2. Investigate changes in estimated glomerular filtration rate (GFR; eGFR), albuminuria, BP, and of CKD and hypertension prevalence from 3 to 5 years post-cancer therapy in CCS at high risk for late kidney and BP outcomes.
3. Investigate whether AKI occurrence during cancer therapy, cardiometabolic risk factors, and/or patient-, condition-, system-, and treatment-specific factors are associated with and/or modify kidney outcomes in CCS at high risk for late kidney and BP outcomes.

A secondary but important aim will be to expand our patient representative committee. We will prioritize ongoing engagement with patient partners to ensure their voices are heard and promoted in research planning. An example of this will be to explore ways of communicating information on long-term kidney and BP outcomes to patients and their families, during cancer therapy.

### Study Design

**Figure 2.**
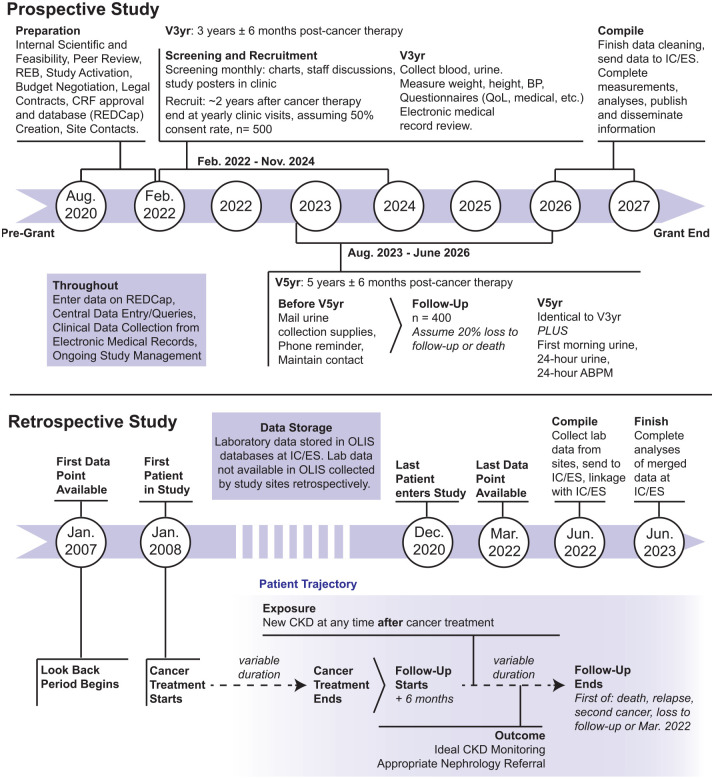
Summary of KINDEST-CCS study timeline and procedures.

This is a prospective cohort study of ~500 CCS followed in pediatric oncology clinics, including “AfterCare” clinics devoted to follow-up of CCS, across 5 Ontario sites over a period of approximately 3.5 years (Figure 2). Study visits will be conducted 3 and 5 years after cancer therapy end, defined as the date of last chemotherapy treatment, radiation, stem cell transplant (SCT) administration (end of therapy for SCT will be 2 years post SCT), or surgery before remission of the patient’s first cancer. The last 5-year follow-up visit will occur in approximately 2026.

### Cohort Participants and Recruitment

The study will enroll CCS who will be ≤18.9 years old at 3 years ± 6 months after cancer therapy completion and received potentially nephrotoxic therapies (eg, platinums, ifosfamide, high dose methotrexate, abdominal or total body radiation, SCT, nephrectomy or other therapies which may be known to cause late kidney and/or BP effects). Patients with pre-cancer CKD diagnoses will be excluded. Given the challenges differentiating underlying hypertension and hypertension secondary to cancer diagnosis, patients with pre-cancer hypertension diagnoses will be included. The definition for cancer therapy end may vary based on cancer treatment group. For example, cancer treatment end will be at 2 years after transplant for SCT patients. Detailed eligibility criteria are outlined in Table 1.

**Table 1.** Prospective Study Inclusion and Exclusion Criteria.

Inclusion Criteria	Exclusion Criteria
• **3 years ± 6 months after therapy for first cancer** • **Received high-risk therapy for first cancer, as defined by the COG (platinums, ifosfamide, high dose methotrexate, abdominal radiation, stem cell transplant, nephrectomy or other therapies which may be known to cause late kidney and/or BP effects)**^a, b^	• Pre-cancer illness CKD (by chart review or by interview with patient/parent/guardian) • Previous kidney transplant • Will be >18.9 years old at date of 3-year follow-up, as determined at screening ~2 years after cancer therapy end

Abbreviations: CKD = chronic kidney disease; COG = Children’s Oncology Group.

a Literature on nephrotoxicity of chemotherapeutic agents (particularly those either known to cause AKI via various mechanisms and/or to cause CKD or hypertension in the long-term) will be reviewed annually and the list of “high-risk chemotherapies” will be reviewed and potentially modified by the principal investigator accordingly.

b Modified lists will be sent to site investigators for agreement and development of a final list. This list may grow as new data/clinical experience become available.

Site coordinators will screen CCS at approximately 2 years post-cancer therapy (using electronic health records and hematology-oncology department databases). Eligible participants will be approached for informed consent either virtually or in person. To support recruitment, study posters with contact information will be advertised in clinics.

### Follow-Up Visits, Data Collection, and Participant Retention

Study visits are scheduled 3- and 5-years post-cancer therapy (Figure 2). For Toronto and Ottawa participants whose 3-year follow-up visit occurs at age >16 years old, conduct of 5-year visit may prove challenging as patients are often transferred to separate adult centers at 18 years of age. At other sites, where pediatric and adult care is provided in the same or nearby institution, the issue of “aging out” as a reason for loss to follow-up (expect < 5% of the total study population) may be mitigated.

Variables of interest collected at 3, 4, and 5 years after cancer treatment are summarized in Table 2. Triplicate measures of weight, height, and BP will be taken at the 3-year visit to calculate adjusted BP percentiles and height-adjusted z-scores.^21^ Sociodemographics, family history of kidney/BP diagnoses, medications, questionnaires,^25,26^ QoL measures,^27,28^ a 5 mL blood sample, and a 30 mL urine sample will also be collected at this time.

**Table 2.** Summary of Visit Schedules and Study Components Occurring After Recruitment at Approximately 2 Years After Cancer Therapy End.

	3-Year Visit	4-Year Contact	5-Year Visit
**Blood sample (5 mL)**	*X*		*X*
**Urine sample (30 mL)**	*X*		*X*
**Phone call to maintain contact**		*X*	
**Mailing of 2 first-morning urine cups and 24-hour urine collection materials** ^a^		*X*	
**Optional 24-hour urine collection** ^b^			*X*
**First-morning urine sample (30 mL)** ^b^			*X*
**Blood pressure (3 measurements)**	*X*		*X*
**Optional 24-hour ABPM**			*X*
**Weight, Height (3 measurements)**	*X*		*X*
**Questionnaires**	*X* ^c^	*X* ^d^	*X* ^c^
**Phone call to maintain contact**		*X*	
**Chart review**	*X*	*X*	*X*

Abbreviations: ABPM = ambulatory blood pressure monitoring.

a Approximately 2 months prior to 5-year visit.

b Within approximately 1 week before the 5-year visit.

c Questionnaires at 3- and 5- year visits include case report form, outcomes of interest, lifestyle questions and PedsQL (including cancer-specific form).

d Questionnaire at 4-year contact include questions regarding new medication, kidney issues, BP issues, or receiving dialysis.

At the 4-year visit, participants will be contacted (phone, email, video conference, in-person during clinic visits per family preference) to maintain correspondence and collect minimal data. Then 2 months before the 5-year visit, participants will be mailed 2 urine cups for first-morning samples and a 24-hour urine collection container with instructions; samples will be collected within a week before 5-year follow-up.

The 5-year visit will mirror the 3-year visit with the addition of: urine samples (2 first-morning and one 24-hour sample) and 24-hour ABPM data for patients 5 years + as per guidelines.^29^ Families will choose whether to have the ABPM device (Ultralight 90217, Spacelabs Med. Inc., Issaquash) placed during the study visit or given instructions on how to place the device and perform ABPM within a week of follow-up. Ambulatory blood pressure monitoring will record wake and sleep BP every 20 and 30 minutes, respectively, and participant diaries will be kept throughout the 24-hour period.^30^

### Biospecimen Handling, Analysis, and Storage

Blood will be centrifuged at 4°C (2000 g × 10 minutes) and serum aliquoted into 1 mL cryovials. Urine will be poured into 2 x 15 mL conical tubes for collection and a dipstick urinalysis. Coordinators will scan specimens into REDCap with barcode readers as they collect/store/ship them for real-time monitoring and quality assurance evaluations. Biospecimens will be stored on-site at −80°C until quarterly shipments to SickKids on dry ice. The central biochemistry lab at SickKids will measure several kidney and cardiovascular markers: serum creatinine (SCr) using isotope-dilution mass spectrometry-traceable assay electrolytes, glucose, cholesterol, triglycerides, serum C-reactive protein, urine albumin to creatinine ratio (ACR), and urine protein to creatinine ratio. Yearly, the Ottawa site biochemistry lab will receive serum shipments for Cystatin C measurement (particle-enhanced turbidimetric immunoassay). Leftover biospecimens will be stored at SickKids at −80°C for repeat measurements as needed and future studies.^31^

### Data Sources and Management

On top of data collected at recruitment and follow-up, clinical data (eg, key medical history, comorbidities, medication lists, recent laboratory values as part of routine care) will be abstracted from patients’ electronic medical records and entered onto paper case report forms (CRFs). Each site will upload de-identified data forms to a secure REDCap database located on the SickKids server. A SickKids data coordinator will enter these data into REDCap and send monthly queries to sites. In previous work, we found central data entry yields fewer errors and reduces study site burden. At the time of participant recruitment, permission will be sought for future data abstraction from patient charts (up to 10 years) to update existing study records.

Only at SickKids, 2 sets of laboratory values will be collected retrospectively, as SickKids only recently (2021) started contributing data to Ontario Laboratories Information System (OLIS): SCr values throughout cancer therapy to define AKI, monthly SCr, urine albumin, and protein values between cancer therapy end and 3-year follow-up and between 3-year and 5-year timepoints.

Following data collection, a database containing all measurements will be sent to ICES for linkage with administrative healthcare data, as done previously.^32-35^ The main patient, system, and treatment factors we will utilize from ICES databases include pre-cancer diagnosis and previously diagnosed cardiac/diabetes/liver disease, dialysis procedures/CKD and/or hypertension diagnoses per administrative healthcare data, birthweight, income quintile, rural *vs.* urban community, and cancer-variables (diagnoses, therapies, SCT, radiation).

### ICES Databases

Administrative health data sources used in the study include The Pediatric Oncology Group of Ontario Networked Information System (POGONIS),^36-38^ a database of clinical information for pediatric oncology patients in the 5 Ontario tertiary care centers, providing rich cancer data^39-43^; Discharge Abstract Database (DAD), Same Day Surgery (SDS), and MOMBABY, documenting administrative, clinical, and demographic characteristics of patients^39,44^; The Ontario Health Insurance Plan (OHIP), ICES-derived Physicians Database (IPDB), and Canadian Organ Replacement Register (CORR), databases used to track physician billing, procedures, and specialist referrals^45,46^; The Registered Persons Database (RPDB), providing demographic information on all Ontarians with health cards^47^ and when they leave the Ontario healthcare system,^48^ providing a method to account for some loss to follow-up; and the OLIS, used to collect laboratory data. A detailed list of data collected from each administrative health data source are provided (Table 3). These databases will also be used for the retrospective study.

**Table 3.** Non-exhaustive Summary of Variables to be Used From ICES Databases and Extracted by Health Records Review for Both the Prospective and Retrospective Studies.

ICES Database	Brief description of how we will use the database	Variables of interest
Pediatric Oncology Group of Ontario Networked Information System (POGONIS) *Available from 1985* *Data collected by all cancer sites*	• Cancer patient identification, sex, age at cancer diagnosis date • Cohort entry date (diagnosis date/date of definitive diagnostic procedure) • Cancer treatment start/end date (first/last treatment given) • Cancer diagnosis type • Treatment type (eg, chemotherapy, radiation, SCTx with details) • Treatment site (hospital) • Death date (linked to other databases, eg, RPDB) • Previous cancer (will be supplemented with Ontario Cancer Registry linkage, not described in proposal)	• Date of Birth • Sex • Pathologically Confirmed Diagnosis • Date of Diagnosis • Radiation Type + Technique • Amount of Radiation Used • Date of Death • Treatment Start/End Date • Date of Receiving Stem Cell • Transplant • Treatment (including “watch and wait” versus palliative versus treatment) • Drug Name and doses for alkylating agents and platinums
Ontario Cancer Registry (OCR) *Available from 1964 +* National Ambulatory Care Reporting System (NACRS) + Cancer Care Ontario Activity Level Reporting (ALR) + Records within the New Drug Funding Plan + OHIP, DAD, SDS below	• Collectively, to supplement POGONIS with treatments (including outpatient and systemic therapies), diagnosis, death, pathologies, and consultation records for patients ≥15 years old who are not found in POGONIS (as per Kitchlu et al^36,37^).	• Age at Diagnosis • Date of Diagnosis • Sex • Diagnosis (and body part), treatments, locations • Relapse, second malignancy
Discharge Abstract Database (DAD) + Same Day Surgery Database (SDS) *Available from 1991*	• Identify hospitalizations and dates, used for comorbidities assessments, diagnoses and procedures (eg, dialysis—for AKI during cancer therapy), demographic information	• Admission/Discharge date • Birth date/Weight/Age • Dialysis, mechanical ventilation, • Radiotherapy, chemotherapy, y/n • Diagnosis/Procedure codes • Region/Education/Residence
MOMBABY database *Available from 1988*	• Contribute to identify admissions prior to cohort entry in infants to evaluate comorbidities • Birth weight	• Birth Date, sex • Birth weight (grams) • Diagnoses
Ontario Health Insurance Plan Claims Database (OHIP) *Available from 1991*	• Identify nephrology consultations through Ontario physician billings • Maintenance dialysis codes—Contribute to end stage renal disease/kidney transplant (pre-cancer CKD- defining) • Define most baseline comorbidities listed in proposal	• Diagnostics Code (includes codes for consultation, dialysis, cardiac, etc.) • OHIP fee code (as above) • Location of OHIP claim • Physician number (for consult type through link with IPDB) • Physician specialty
Registered Persons Database (RPDB) *Available from 1991*	• Date of last contact (and OHIP coverage end date) • Birth date • Death date (with POGONIS) • Socioeconomic status and rural variables	• Birth date • Death date • Neighborhood Income Quintile • Rurality Index • Date of last contact
Canadian Organ Replacement Registry (CORR) *Available from 1981*	• Past kidney transplant, dialysis • Kidney transplant, maintenance dialysis (for CKD definition, pre-cancer)	• Kidney Transplant • Death date • Hemodialysis/Peritoneal dialysis
ICES Physician’s Database (IPDB) *Available from 1991*	• Physician specialty for visits (eg, oncology care, AfterCare, primary care; nephrology)	• Physician specialty (first, second, etc.) • Primary care (yes/no)
Ontario Laboratory Information System (OLIS) Database *Available from 2007*	• Serum creatinine, cystatin C, urinary protein/albumin, vitamin D, hemoglobin, parathyroid hormone measures • Ordering center, provider, and lab destination • ***For prospective study*:** Goal is mainly to identify AKI during cancer therapy, as we have previously defined, using serum creatinine criteria of the KDIGO definition. OLIS will provide serum creatinine data to define AKI during cancer therapy for all sites except SickKids, which has not yet contributed to OLIS. ***For the retrospective study*** (need labs as far back as 2007), OLIS data available for sites in Kingston, Hamilton, London, and Ottawa from as far back as 2015, 2014, 2013 and 2016, respectively.	• Birth Date • Destination Lab • Measure type (eg, albumin, protein) • Measure value (+ ref range, units) • Ordering Facility / Provider
Variables to be collected by site staff via chart abstraction	Labs above and dates; whether inpatient or outpatient. *Prospective study*: Baseline and study visit medical, medication and family history characteristics; inclusion and exclusion criteria (including relevant chemotherapies received); routinely collected labs above performed closest to the study visits. *At SickKids only*: serum creatinine values throughout cancer therapy to define AKI and monthly serum creatinine and urine albumin/protein performed during routine care from cancer therapy end to date of first study visit. *Retrospective study*: Lab values described above will need to be collected by site research staff during the following time periods (years not available in OLIS provincial database): Kingston: 2007-2014 Hamilton: 2007-2013 London: 2007-2012 Ottawa: 2007-2015 SickKids: 2007-2020	• Destination Lab • Measure type (eg, albumin, protein) • Measure value (+ ref range, units) • Ordering Facility / Provider • CKD y/n, adherence y/n (from measures) • Together with other databases, determine if inpatient or outpatient lab test.

Abbreviations: POGONIS = Pediatric Oncology Group of Ontario Networked Information System; SCTx = stem cell treatment; OCR = Ontario Cancer Registry; NACRS = National Ambulatory Care Reporting System; OHIP = Ontario Health Insurance Plan; DAD = Discharge Abstract Database; SDS = Same Day Surgery Database; AKI = acute kidney injury; CKD = chronic kidney disease; IPDB = ICES Physician Database; RPBD = Registered Persons Database; OLIS = Ontario Laboratory Information System; CORR = Canadian Organ Replacement Registry; KDIGO = Kidney Disease Improving Global Outcomes; ALR = activity level reporting.

### Outcome and Exposure Definitions

The primary outcomes in this study are post-cancer therapy CKD and hypertension. Chronic kidney disease will be defined as low eGFR [eGFR<90 mL/min/1.73 m^2^; Grade 2 CKD or worse] or albuminuria [ACR >3 mg/mmol] per KDIGO guidelines^19^ (Table 4). Grade 3 CKD will also be described. In this prospective study, GFR will be estimated using a validated equation including SCr and Cystatin C.^49,50^ In secondary analyses, 5-year follow-up visit GFR will be defined using the creatinine clearance measured from 24-hour urine collection. Hypertension will be defined according to American Academy of Pediatrics (AAP) using height-, sex-, and age-adjusted BP percentile tables^21,29^ (Table 5). At the 5-year study visit the presence of either ambulatory hypertension or masked hypertension will be the main ABPM outcome; however, presence/absence of any ABPM abnormality and the prevalence of individual ABPM component abnormalities will also be treated as outcomes (Supplementary Table 2).

**Table 4.** Simplified Version of the KDIGO Definition for Chronic Kidney Disease (CKD) Used in This Study, Based on Low Estimated Glomerular Filtration Rate (eGFR) or Albuminuria (ACR).

CKD Grading	Estimated glomerular filtration rate (eGFR)		Albumin-to-creatinine ratio (ACR)	Time Frame (only retrospective study)
Grade 1	≥90 mL/min/1.73 m^2^	OR	<3 mg/mmol	For ≥3 months
Grade 2 or worse	<90 mL/min/1.73 m^2^		≥3 mg/mmol	
Grade 3 or worse	<60 mL/min/1.73 m^2^		≥30 mg/mmol	

Notes: Albuminuria= ≥3 mg/mmol; Grade 1 CKD = known kidney problems, but eGFR is still normal and there is no significant albuminuria.

Abbreviations: KDIGO = Kidney Disease: Improving Global Outcomes Guidelines; CKD = chronic kidney disease; eGFR = Estimated glomerular filtration rate; ACR = Albumin-to-creatinine ratio.

**Table 5.** American Academy of Pediatrics Classification of Blood Pressure According to Casual Blood Pressure Measures Guidelines.

	Children 1 to <13 years old	Children ≥ 13 years old
Normal	<90th percentile	<120/< 80 mmHg
Elevated blood pressure	≥90th to <95th percentile or 120/80 mmHg to <95th percentile (whichever is lower)	120/< 80 to 129/< 80 mmHg
Stage 1 hypertension	≥95th to <95th percentile + 12 mmHg or 130/80 to 139/89 mmHg (whichever is lower)	130/80 to 139/89 mmHg
Stage 2 hypertension	≥ 95th percentile + 12mmHg or ≥140/90 mmHg (whichever is lower)	≥140/90 mmHg

Abbreviations: mmHg = millimeters of mercury.

*Changes* in eGFR, albuminuria, and BP percentile from the 3-year to the 5-year visits, *persistent* CKD, and *persistent* hypertension at 3- and 5-year visits, and *new* CKD or hypertension at the 5-year visit will be recorded. Aim 3 outcomes will be the same as those described above.

Study exposures of interest pertain mainly to aim 3: AKI during cancer therapy and presence of cardiometabolic risk factors. Acute kidney injury will be defined based on KDIGO guidelines for AKI definition, as a ≥50% SCr rise throughout cancer therapy, from baseline, as previously described.^19,51,52^ Cardiometabolic risk factors will be ascertained at the study visits and will include glucose, body mass index (BMI) z-score (with secondary analyses using overweight/obesity classifications), non-fasting lipid profile (triglycerides, high- and low-density lipoprotein cholesterol), and C-reactive protein.

### Statistical Analysis

Multivariable analyses described below will include variables of cancer diagnosis classified by a previously described classification system,^53^ age at diagnosis, birthweight, sex (gender), income quintile, rural *vs.* urban, and baseline cardiac, diabetes or liver disease, unless stated otherwise. Sex-stratified analyses will also be conducted. Effect estimates will be described with 95% confidence intervals.

Three- and 5-year prevalence of CKD, hypertension, all categorical 5-year ABPM outcomes, and continuous outcome measures (eg, eGFR; BP percentile) will be calculated. We will compare 3- *vs.* 5- year CKD and hypertension prevalence using the McNemar test, calculate 3- to 5-year eGFR, ACR, and BP percentile change and proportions of participants with persistent CKD, hypertension and status change. We will use mixed effect models (for repeat measures) to evaluate adjusted associations of AKI and cardiometabolic measures with 3- and 5-year outcomes to evaluate if they modify trajectories by including interaction terms for these measures with time in models. We will also evaluate univariable and multivariable associations of AKI and cardiometabolic measures (lipids, glucose, BMI) with individual 3- and 5-year binary outcomes (eg, CKD) using log-binomial regression and continuous outcomes using linear regression (eg, eGFR). In exploratory analyses, we will determine if analyses above differ by treatment groups (eg, platinums, radiation, etc.), treatment combinations (eg, receiving cisplatin and radiation), and diagnosis classification^53^ in univariable analyses, and if event rates allow, in multivariable analyses. Sample size justification is provided in Supplementary Information.

## Retrospective Study: CKD and Hypertension Screening, Complications Monitoring, and Administrative Data Validation in Childhood Cancer Survivors

### Aims and Objectives

In a retrospective cohort of Ontario CCS treated for cancer between 2008 and 2020, we will evaluate:

1a. Chronic kidney disease screening, complications monitoring (Vitamin D, parathyroid hormone, hemoglobin, SCr, proteinuria) and nephrology referral;
1b. Hypertension screening, complications monitoring (BP follow-up measures, 24-hour BP monitoring, echocardiogram) and nephrology referral.
2. The validity of several pediatric specific CKD and hypertension administrative algorithms
3. Chronic kidney disease and hypertension incidence in the ~10 years after cancer therapy.
4. Whether patient, disease/treatment or system factors are associated with each of the above outcomes.

### Study Design and Cohort

The retrospective multi-center cohort study consists of approximately 5,000 CCS treated for cancer across 5 Ontario sites between January 2008 and December 2020. Hypertension outcome analyses will include SickKids patients only (approximately 2500 CCS) due to data collection feasibility. The inclusion and exclusion criteria are shown in Table 6. Briefly, all children treated for cancer during the study period, who survive at least 6 months post-cancer treatment end will be included. Figure 2 shows that cohort entry date is at cancer diagnosis (first patient enrolled January 2008, allowing for a 1-year look-back period to January 2007 for labs and baseline characteristics from administrative healthcare data); only patients surviving ≥6 months from cancer therapy end will be evaluated for outcomes (eg, adequate CKD monitoring and nephrology referral); those dying or censored before then will be described to ascertain potential biases. Childhood cancer survivor with CKD at <6 months post-therapy end will be classified as CKD at follow-up start. The last patients to enter the cohort will be those with diagnosis occurring on or before December 2020 (Figure 2; Table 6) to allow at least 1.5 years of data available from administrative healthcare data after diagnosis (date of last data point to be used: March 2022). Patients will be censored on March 2022, date of death, second cancer, relapse, or loss to follow-up—whichever comes first.^54^ In sensitivity analyses, we will consider including second cancer and relapse as binary variables in the multivariable analyses described below, rather than as censoring events (not described further).

**Table 6.** Retrospective Study Inclusion and Exclusion Criteria.

Inclusion Criteria	Exclusion Criteria
All patients treated for cancer^a^	Invalid/missing health number, birth date or sex
≤18 years old at cancer diagnosis, Jan 1, 2008-Dec 31,2020.^b^	Children surviving <6 months after treatment end

a The Pediatric Oncology Group of Ontario Networked Information System (POGONIS) will be used to identify these patients, as previously described.

b Dates selected to ensure necessary administrative health data is available as far back as 2007 and at least 1-2 years post-cancer diagnosis.

### Data Sources

Much of the data used will be obtained from ICES databases (described in Table 3). One important exception will be laboratory data; all sites will collect labs onto a paper CRF as far back as 2007 (for entry into REDCap at the study coordinating center) or receive spreadsheets of desired laboratory variables from Health Records departments (depending on site approved processes). If unavailable, these data may then be supplemented by OLIS. Dates of laboratory tests availability will vary by site (Table 3). Other variables of interest (eg, cancer diagnosis, treatment variables) available at sites will be abstracted from patient’s electronic medical records at all sites and entered onto paper CRFs, to be later entered into REDCap. Outpatient BP values and echocardiogram data will be collected at SickKids only. Following data collection, study data will be sent to ICES for linkage with administrative healthcare databases.

### Exposures and Outcomes

Aim 1 primary outcomes include ideal CKD and hypertension monitoring and appropriate nephrology referral. As shown in Table 4, ideal CKD monitoring can change yearly depending on the CKD severity, as per KDIGO guidelines.^19^ Ideal CKD monitoring in CCS will be categorized as yes/no according to CKD stage as described in Table 7. Appropriate nephrology referral will be defined as a referral within 1 year of CKD appearance or within 1 year of CKD progression (≥3 months of CKD Grade worsening or 25% eGFR drop or eGFR <60 mL/min/1.73m^2^ or proteinuria ≥50 mg/mmol or albuminuria ≥30 mg/mmol). Ideal hypertension monitoring will be defined as performance of a 24-hour ABPM and echocardiogram within 6 months of hypertension appearance, as defined in Table 5 based on age-, height- and sex- percentiles. Appropriate hypertension nephrology referral will be defined as nephrology referral within 6 months of hypertension appearance.^21^

**Table 7.** Simplified Summary of Ideal Chronic Kidney Disease (CKD) Monitoring (in Children, Combined Recommendations From KDIGO and the KDOQI Quidelines Are Used, as Recommended).

CKD Categories	Monitoring Targets^a^	Laboratory Measures				
		Vitamin D	Parathyroid Hormone	Hemoglobin	Serum Creatinine (eGFR)	Urine Protein (ACR)
Grade 1	None indicated					
Grade 2	≥1 measures at least once after Grade 2 CKD appearance	**X**	**X**	**X**		
	≥1 measure(s) within 2 years of an abnormal value	**X**	**X**	**X**		
	≥yearly measures after Grade 2 CKD				**X**	**X**
Grade 3	≥1 measure(s) within 1 year of Grade 3 CKD appearance	**X**	**X**	**X**		
	≥1 measure(s) within 1 year of an abnormal value	**X**	**X**	**X**		
	≥yearly measures after Grade 3 CKD appearance			**X**	**X**	**X**

Abbreviations: KDIGO = Kidney Disease: Improving Global Outcomes Guidelines; KDOQI = Kidney Disease Outcomes Quality Initiative; eGFR = estimated glomerular filtration rate; ACR = albumin-to-creatinine ratio.

a Patients with no CKD will not be labeled as requiring monitoring. Patients with CKD will only be classified as “no” for ideal monitoring if enough time has passed. This allows CKD Grade (and ideal monitoring) to change.

The primary exposures for aim 1 are CKD and hypertension. CKD will be defined in 2 ways, detailed in Table 4: Grade 2 CKD or worse (binary; and also staged by CKD severity); and a stricter definition requiring ≥2 outpatient, abnormal eGFRs or albuminuria (or proteinuria) results, greater than 3 months apart, with no normal results in between.^55-57^ eGFR will be calculated with only SCr if cystatin C is unavailable; a sensitivity analysis for patients with both analytes available will be conducted. Should height be unavailable (for pediatric GFR estimation), validated height-independent eGFR equations will be used.^50^,^58-60^ Hypertension will be defined as 2 or more consecutive hypertensive values, defined in Table 5 based on age-, height- and sex- percentiles, on 2 separate days. Similar to other outcomes, patient-, disease/treatment-, and system-related factors will be evaluated. CKD and hypertension will also be defined in this way to attain aim 3, describing the incidence of CKD and hypertension in the ~10 years after cancer therapy.

Aim 2 (validation of administrative healthcare data for identifying CKD and hypertension) outcomes will be CKD and hypertension, defined using laboratory data and BP measures, respectively. These outcomes will be defined using the strict criteria described above (ie, reference standard method) and considered the “gold standard.” The diagnostic test will be algorithms based on diagnosis and procedure codes available from ICES databases (described in previous work). These CKD and hypertension algorithms in the ICES databases will be evaluated for their ability to identify or detect CKD and hypertension defined by reference standard-methods.^61,62^

### Statistical Analysis

We will calculate yearly proportions of patients with CKD and hypertension who undergo ideal monitoring, and appropriate nephrology referral, separately. We will also evaluate individual CKD and hypertension monitoring components (eg, Vitamin D measurement). Yearly measurement rates of eGFR and urine protein measures from the end of therapy will also be calculated. We will compare characteristics and monitoring in CCS with *vs.* without CKD and hypertension to investigate factors related to variation in clinical practice. Using log-binomial regression (with generalized estimating equations, accounting for within subject correlation), we will estimate the yearly change in ideal monitoring. We will use multivariable log-binomial regression with generalized estimating equations to yield adjusted rate ratios of patient, disease, treatment, and system covariates for ideal monitoring. Analyses will be repeated, adjusting for time as well as provider-level and center-level clustering in monitoring practices.

Various diagnosis and procedure code-based algorithms for CKD and hypertension will be evaluated for detecting reference-standard-based CKD and hypertension (Aim 2). Algorithms will be evaluated for validity and level of agreement with the following measures: sensitivity, specificity, positive predictive value (PPV), negative predictive value (NPV), kappa (κ) statistic, and area under the receiver operating characteristic (ROC) curve.

We will use a sub-distribution hazards model accounting for competing risk of death (Fine and Gray method)^63^ to estimate cumulative incidence (including at pre-defined time intervals: 1, 3, 5, 7, 10 years) and generate cumulative incidence function curves to understand burden and time of CKD and hypertension onset (aim 3). Censoring will occur at system leave date (ie, emigration), relapse, second malignancy, or follow-up end.^54,64^ Sample size calculation is provided in supplementary information.

### Timeline and Knowledge Translation (KT) Plans

Table 8 shows the overall timeline. The benefit gained from these 2 studies will be magnified by a thoughtful and iteratively planned KT strategy. Knowledge dissemination at meetings and in publications will target kidney, oncology, and general pediatrics audience. Early on, we will engage kidney, oncology and pediatric stakeholder and patient groups (eg, Canadian Society of Nephrology; Canadian Cancer Society; Pediatric Oncology Group of Ontario; Canadian Pediatric Society; Cancer Care Ontario), on their research missions. We hope to foster partnerships between these groups (eg, nephrology groups with pediatric oncology groups) by identifying common goals for CCS cardiovascular and kidney health. In year 1, we will begin creating a larger patient advisory board and approach international investigators performing similar work, to collaborate on common CCS health goals. Furthermore, patient and provider surveys will explore perspectives, practice gaps, and research priorities to inform KT for the foreseeable future.

**Table 8.** Study Timeline Over 6 Years, From Pre-study Start to April 2027.

Item	2020-2021 Pre	2021-2022 Year 1		2022-2023 Year 2		2023-2024 Year 3		2024-2025 Year 4		2025-2026 Year 5		2026-2027 Year 6	
SickKids Research Ethics Board (REB), then other sites + contracts													
Develop forms, REDCap, validity/reliability													
**Aim 1**: Train sites staff, recruit, 3- and 5-year visits													
**Aim 1**: Finish lab measures, ICES analysis plan, list to ICES													
**Aim 1**: SickKids data cleaning, ICES linkage, analyses													
**Aim 1:** Initial manuscripts													
**Aim 2**: REB, ICES analysis plan													
**Aim 2**: Get supplemental institution lab data, clean data													
**Aim 2**: Institution lab data linkage to ICES data and analyses													
**Aim 2:** Initial manuscripts													
Monthly KDT, bi-monthly investigator team meetings													
Core Patient partner/Knowledge user quarterly meetings													
Knowledge Translation specific timeline (excluding manuscripts)													
Identify stakeholder/policy maker groups to contact													
Stakeholder/policy maker meetings (year 1 and throughout)													
Identify patient partners for larger group													
Create larger patient partner group, regular meetings													
Contact other country common-goal groups/societies/cardiology													
Create links between kidney-cancer groups													
Patient, knowledge user, stakeholder surveys													
In-person/telecon planning meeting (trials, guideline, knowledge translation)													
Present at cancer, kidney, pediatrics scientific meetings													
Present to patient, stakeholder, Knowledge user groups													
Plan next grants													

Abbreviations: REB = research ethics board; KDT = Kidney, Dialysis and Transplantation Research Program.

At years 3 and 5, we will host videoconference meetings with investigators, patients, stakeholders, and policy representatives to discuss progress, surveys, and update/unify priorities. We also plan to hold working groups to discuss action plans in response to actual and potential findings (eg, Prospective study: If hypertension is common, how to proceed? Clinical trial? Guideline changes/ implementation trial? What are success measures?), discuss steps to impact CCS guidelines based on burden findings from the prospective study and practice gaps from the retrospective study. We will know normative practice patterns, gaps, and associated factors of CKD and CKD-related complications. Our cohort, data and biobank will open countless avenues of novel cross-disciplinary research endeavors and training. Our team will be ideally placed to inform and apply CCS kidney and BP guidelines within the greater context of CCS health and maximize patient and system beneficial impact from the knowledge we generate.

## Discussion

While it is known that CCS are at increased risk of adverse kidney effects, limitations in our understanding of long-term effects of cancer therapy may track as late complications. CKD and hypertension burden in the first 5 years after cancer therapy is unclear, as are current recommendations on follow-up for CCS.

The involvement of all major cancer centers in Ontario, facilitating diverse patient recruitment including a spectrum of therapies is a certain strength of this study. Our use of robust definitions for CKD, hypertension, and AKI than currently available will increase the validity of results and applicability to current clinical practice. To our knowledge, this will be the first study to implement 24-hour ABPM, gold-standard for detection of hypertension in a large cohort of CCS, permitting comprehensive diagnosis of types of hypertension.^65^

A major goal is to describe current management of CKD and hypertension complications in CCS and establish practice gaps in this patient population. When consulting best practices in managing CCS at higher risk for CKD, clinicians may turn to KDIGO^19^ or COG.^9,18^ KDIGO provides management objectives for patients with or at risk of CKD, but is not adapted to the CCS health context, whereas COG kidney guidelines are used internationally to identify and manage cancer therapy effects in CCS, but does not provide actionable recommendations on managing kidney complications.^18,19^ We believe that data generated from this study may enable improvements in both pediatric oncology and of overall CKD and hypertension guidelines, especially with a targeted KT strategy.

Another strength is our assessment of whether cancer-, patient-, therapy-, and system-specific factors influence kidney and BP outcomes or screening and follow-up practices when treating CCS.^66-68^ Mothers experiencing high stress and/or with residences far from neonatal clinics were less likely to attend follow-up appointments.^68^ If specific barriers are identified, an informed, targeted approach may lead to improved quality of care and health outcomes. Our aim to validate administrative CKD and hypertension definitions in this population would dramatically enhance understanding of disease burdens in this population, using administrative data.

Both studies have limitations. In the prospective study, a proportion of CCS will have a cancer relapse, which may prove difficult to discern between worsening disease- and treatment-associated kidney dysfunction. Survivor bias will be an issue when analyzing risk factors for treatment-associated CKD and hypertension. We can compare survivors’ *vs.* non-survivors’ characteristics, conduct sensitivity analyses to assess the impact of differential loss to follow-up, and if needed, propensity scores (eg, for AKI) can be used in multivariable analyses to mitigate these biases. There may be small sample sizes for subgroup analyses of specific treatments; however, our patient partners stressed these subgroup analyses are priorities.

We acknowledge including study visits before 3 years would provide insight on early post-therapy development of CKD and hypertension. We chose 3-year follow-up for cohort entry to improve feasibility/cost and allow time for resolution of immediate therapy-related effects, while capturing patients early enough to identify early detection and intervention opportunities.^15^ By design, conclusions in the prospective study only apply to high-risk CCS; however, this is a priority population for evidence-based guideline development and will benefit the most from early CKD and hypertension diagnosis and intervention. Moreover, our high-risk groups definitions are aligned with risk groups delineated by the COG long-term follow-up guidelines, which will enhance future KT. One potential issue is 3 and 5-year prospective study event rates may be lower than expected; however, previous work^69^ demonstrates high event rates and including only high-risk CCS makes this problem unlikely. If low event rates are found, this will direct future research on the timing of CKD and hypertension onset in CCS. We may not see much change between the 3- and 5-year outcomes, but this first-of-its-kind evaluation of sustained kidney or BP abnormalities will establish rate-of-change data for future research. ABPM will only be done at the 5-year visit to reduce attrition bias. Similar to most albuminuria studies, ACR is susceptible to postural proteinuria and overestimation. This is a challenge in all pediatric studies evaluating proteinuria. We attempt to mitigate this by providing participants with instructions to effectively perform first-morning urine prior to the 5-year visit. We acknowledge that the study aim is descriptive in measuring changes in the outcomes.

In the retrospective study, practice drift may be evident. However, we do not expect this as COG guidelines have not changed significantly in recent editions in 2013.^18,70^ We will evaluate pre *vs.* post 2013 eras and will include this variable in analyses if differences are found. The retrospective study is susceptible to surveillance bias; sicker patients are tested more often for CKD, thus included in analyses. We will use this large database to calculate CKD incidence using routinely collected data, but because of ascertainment bias concerns, this was not the primary goal of this study. A possible challenge for the retrospective study relates to using tests (eg, hemoglobin) to determine if complication monitoring occurs; it will not be possible to know if a test is specifically done to monitor for CKD. For atypical tests (eg, parathyroid hormone), this is likely not an issue. We also may be unable to infer the specific intentions of clinicians when ordering the test, nor if treatments were given in response to abnormal test results; we can only know whether or not tests were done. In future work, we may determine if treatments were given in response to test abnormalities by incorporating healthcare provider surveys with prospective data collection.

## Conclusions

Guidelines to monitor CKD^19^ and hypertension^21^ exist but are not adapted to the natural history of CKD and hypertension specifically in CCS. We aim to prospectively study 3-year and 5-year CKD and hypertension outcomes and retrospectively determine whether appropriate screening practices are used for kidney outcomes in CCS. With this information we will be able to identify practice gaps within the current CCS healthcare context in Ontario and inform modifications to current guidelines for monitoring late kidney complications in CCS.^18,19,21,71,72^

## Supplemental Material

sj-docx-1-cjk-10.1177_20543581221130156 – Supplemental material for KIdney aNd blooD prESsure ouTcomes in Childhood Cancer Survivors: Description of Clinical Research Protocol of the KINDEST-CCS Study Click here for additional data file. Supplemental material, sj-docx-1-cjk-10.1177_20543581221130156 for KIdney aNd blooD prESsure ouTcomes in Childhood Cancer Survivors: Description of Clinical Research Protocol of the KINDEST-CCS Study by Adree Khondker, Michael Groff, Sophia Nunes, Carolyn Sun, Natasha Jawa, Jasmine Lee, Vedran Cockovski, Yasmine Hejri-Rad, Rahul Chanchlani, Adam Fleming, Amit Garg, Nivethika Jeyakumar, Abhijat Kitchlu, Asaf Lebel, Eric McArthur, Luc Mertens, Paul Nathan, Rulan Parekh, Serina Patel, Jason Pole, Raveena Ramphal, Tal Schechter, Mariana Silva, Samuel Silver, Lillian Sung, Ron Wald, Paul Gibson, Rachel Pearl, Laura Wheaton, Peter Wong, Kirby Kim and Michael Zappitelli in Canadian Journal of Kidney Health and Disease
