# Supplementary material for: KIdney aNd blooD prESsure ouTcomes in Childhood Cancer Survivors: Description of Clinical Research Protocol of the KINDEST-CCS Study
Source: Can J Kidney Health Dis. 2022 Oct 27;9:20543581221130156. doi: 10.1177/20543581221130156 (PMC9618744; doi:10.1177/20543581221130156)
Supplement: sj-docx-1-cjk-10.1177_20543581221130156 – Supplemental material for KIdney aNd blooD prESsure ouTcomes in Childhood Cancer Survivors: Description of Clinical Research Protocol of the KINDEST-CCS Study [file sj-docx-1-cjk-10.1177_20543581221130156.docx]

**Supplementary Information**

**KI**dney a**N**d bloo**D** pr**ES**sure ou**T**comes in **C**hildhood **C**ancer **S**urvivors:

Description of study protocol of the KINDEST-CCS study

*Adree Khondker^1,2^, *Michael Groff^1,3^, Sophia Nunes^1^, Carolyn Sun^1^, Natasha Jawa^4^, Jasmine Lee^1^, Vedran Cockovski^1^, Yasmine Hejri-Rad^1^, Rahul Chanchlani^5^, Adam Fleming^6^, Amit Garg^7^, Nivethika Jeyakumar^8^, Abhijat Kitchlu^9^, Asaf Lebel^4^, Eric McArthur^10^, Luc Mertens^11^, Paul Nathan^12^, Rulan Parekh^1,4^, Serina Patel^13^, Jason Pole^14^, Raveena Ramphal^15^, Tal Schechter^12^, Mariana Silva^16^, Samuel Silver^17^, Lillian Sung^12^, Ron Wald^18^, Paul Gibson^14^, Rachel Pearl^19^, Laura Wheaton^16^, Peter Wong^19^, Kirby Kim^20^, Michael Zappitelli^1,4^.

** The first two authors have contributed equally to this work and are co-first authors.*

^1^Child Health Evaluative Sciences, The Hospital for Sick Children, Toronto, Ontario, Canada

^2^Temerty Faculty of Medicine, University of Toronto, Toronto, Ontario, Canada

^3^Department of Epidemiology and Biostatistics, Schulich School of Medicine and Dentistry, The University of Western Ontario, London, Ontario, Canada.

^4^Division of Nephrology, Department of Pediatrics, The Hospital for Sick Children, Toronto, Ontario, Canada

^5^Department of Pediatrics, McMaster Children's Hospital, Hamilton, Ontario, Canada

^6^Department of Pediatric Hematology/Oncology, McMaster Children's Hospital, Hamilton, Ontario, Canada

^7^Department of Medicine, London Health Sciences Centre Research Inc., London, Ontario, Canada

^8^Institute of Clinical Evaluative Sciences Western, London, Ontario, Canada

^9^Department of Medicine/ Nephrology, University of Toronto, Toronto, Ontario, Canada

^10^Institute for Clinical Evaluative Sciences, Toronto, Ontario, Canada

^11^Division of Cardiology, The Labatt Family Heart Center, The Hospital for Sick Children, Toronto, Ontario, Canada

^12^Division of Hematology/Oncology, Department of Pediatrics, The Hospital for Sick Children, Toronto, Ontario, Canada

^13^Department of Pediatric Hematology/Oncology, Children's Hospital of Western Ontario, London, Ontario, Canada

^14^Pediatric Oncology Group of Ontario, Toronto, Ontario, Canada

^15^Department of Pediatrics, Children's Hospital of Eastern Ontario-Ottawa Children's Treatment Centre, Ottawa, Ontario, Canada

^16^Department of Pediatrics, Kingston Health Sciences Centre, Kingston, Ontario, Canada

^17^Department of Medicine/Nephrology, Queen’s University, Kingston, Ontario, Canada

^18^Unity Health Toronto, Toronto, Ontario, Canada

^19^William Osler Health Centre, Brampton, Ontario, Canada

^20^Patient Partner, The Hospital for Sick Children, Toronto, Ontario, Canada.

**Corresponding Author’s contact information**

Michael Zappitelli

The Hospital for Sick Children, Peter Gilgan Centre for Research and Learning

686 Bay St. 11^th^ Floor. Room 11.9722. Toronto, Ontario. M5G 0A4.

Telephone 416-813-7654 X304077; email [Michael.Zappitelli@sickkids.ca](mailto:Michael.Zappitelli@sickkids.ca)

*Prospective Study Sample Size Justification*

The sample size of 500 participants at the 3-year post-cancer therapy visit and assumption of 20% attrition (400 participants remaining) was determined based on published estimates of yearly cancer diagnoses in Ontario, published 5-year mortality rates and previous retention rates in our studies. This sample size ensures adequate study precision (no more than ± 5%) for our primary outcomes (CKD, hypertension). Our previous work strongly suggests that 3-year prevalence of CKD and hypertension as per our definitions will be at least 30% and 25%, respectively. This sample size also allows for multivariable analyses and selected subgroup analyses to be performed.

*Retrospective Study Sample Size Justification*

We estimate, based on prior publications, that approximately 5000 CCS will be included in this study^1-5^. Given our use of clinical routine care data (as opposed to systematically study-measured data), if we estimate only ~10% develop CKD (n=500), and even if only 10% of patients have ideal monitoring, we can estimate monitoring with ±2.6% precision, with adequate sample size for planned multivariable analyses. Approximately 2500 patients will be included in hypertension monitoring analyses, given our use of clinical routine care data, if we estimate only ~25% develop hypertension (n=625), even if only 10% of patients have ideal monitoring, we can estimate this with ±2.9% precision. Power calculations (assuming α= 0.05) also revealed adequate level of precision for sensitivity and specificity calculations (aim 2) and CKD and hypertension incidence analyses (aim 3).

**Supplementary Tables and Figures**

**Supplementary Table 1.** Key roles and expertise of the KINDEST-CCS study team members.

| **Name** | **Role and Description** |
| --- | --- |
| Michael Zappitelli | **Nominated Principal Applicant**:  M.Z. is a pediatric nephrologist and clinician scientist at SickKids, and associate professor at the University of Toronto. He is an international expert on child acute kidney injury outcomes, glomerular filtration rate and multi-center pediatric cohort studies. M.Z. will supervise all aspects of the grant including the core team (Lee, Cockovski, Wang). |
| **Nephrology/Epidemiology Co-Applicants** | |
| Rahul Chanchlani | R.C. is a pediatric nephrologist and assistant professor at McMaster Children’s Hospital. He is a young investigator, key knowledge user with ICES and expert in child kidney research. R.C. will assist in interpreting results of 24-hour ambulatory blood pressure monitoring. |
| Amit Garg | A.G. is an adult nephrologist, scientist and director of ICES Kidney, Dialysis and Transplantation Research Program. He is a kidney initiative leader with extensive experience in ICES research. A.G. co-chaired the development of Kidney Disease: Improving Global Outcomes living kidney donor guidelines and is a key liaison with the Canadian and international kidney community. He will provide input on design and methods of the studies. |
| Ron Wald | R.W. is an adult nephrologist at the University of Toronto with expertise in clinical trials, epidemiological, and ICES studies. He is also the co-chair of the ICES Kidney, Dialysis and Transplantation Research Program. |
| Samuel Silver | S.S. is an adult nephrologist and new investigator. He is a quality of kidney care research expert and member of the ICES Kidney, Dialysis and Transplantation Research Program. |
| Rulan Parekh | R.P. is the associate chief of Clinical Research at SickKids and Canada Research Chair in chronic kidney epidemiology. R.P. holds expertise in ICES and cohort research and will provide local support to the nominated principal applicant. |
| Abhijat Kitchlu | A.K. is an adult nephrologist at the University of Toronto and new investigator. A.K. contributed ICES knowledge to capture older children in the current study protocol. He will contribute to defining non-kidney (cancer, cardiovascular disease, etc.) covariates and outcomes and future knowledge translation. |
| **ICES analyst/project Co-Applicants** | |
| Nivethika Jeyakumar | N.J. is an ICES analyst, experienced with using ICES kidney data in prospective and retrospective observational kidney research. She will lead ICES-level grant coordination, dataset creation plan assistance, ICES analyses, and ICES Kidney, Dialysis and Transplantation Research Program conference calls. |
| Eric McArthur | E.M. is an ICES analyst and statistician with years of experience working with ICES kidney data. E.M. will supervise ICES data analyses and will plan future data use. |
| **Oncology Co-Applicants** | |
| Jason Pole | J.P. is a professor at the Centre for Health Services Research (University of Queensland, Australia) with a background in epidemiology. He is an adjunct scientist with the Pediatric Oncology Group (POGO) with expertise in ICES data, variable validation, and definitions specific to pediatric cancer. J.P. will provide key input on pediatric cancer research and connecting with other relevant organizations. |
| Paul Nathan | P.N. is a pediatric oncologist and senior scientist at SickKids with expertise in childhood cancer survivor research and knowledge translation. He also has expertise in POGO and ICES data, variable validation, and outcomes. He is also a strong POGO, Children’s Oncology Group and other cancer groups liaison. P.N. is the solid tumour patient site investigator in Toronto and solid tumour expertise lead. |
| Lillian Sung | L.S. is a pediatric oncologist at SickKids with expertise in research and knowledge translation, clinical trials, and guidelines in cancer. L.S. is a multi-center researcher expert and strong POGO, Children’s Oncology Group and other cancer groups liaison. L.S. will provide key input on feasibility and utilization of results for knowledge translation. |
| Tal Schechter | T.S. is a pediatric hematologist-oncologist at SickKids and expert in stem cell transplant late childhood cancer survivor outcomes. She is a strong liaison for Children Oncology Group’s stem cell transplant groups. T.S. is the Toronto stem cell transplant site investigator and stem cell transplant expertise lead. |
| Adam Fleming | A.F. is a pediatric hematologist-oncologist and oncology clinics leader at McMaster University. A.F. is an expert in solid tumours and neuro-oncology. A.R. is the site investigator in Hamilton. |
| Raveena Ramphal | R.R. is a pediatric hematologist-oncologist at the University of Ottawa with expertise in adolescent oncology. R.R is the site investigator in Ottawa. |
| Mariana Silva | M.S. is a pediatric hematologist-oncologist and professor at Queen’s University. M.S has cancer education expertise and is a strong liaison for several key stakeholder groups. M.S. is the site investigator in Kingston. |
| Serina Patel | S.P. is a pediatric hematologist-oncologist at Western University. S.P is a new investigator with interest in late effects and fertility and the site investigator in London. |
| **Cardiology Co-Applicant** | |
| Luc Mertens | L.M. is a pediatric cardiologist and head of SickKids Echocardiography and Research Lab with expertise on cardiovascular disease in children, cardiometabolic risk and evaluation of cardiovascular damage. L.M. will be key to knowledge translation and interpretation of results as part of the integrated knowledge translation plan. He will also be a liaison with cardiology societies. |
| **Collaborators** | |
| Rachel Pearl | R.P. is a general pediatrician and nephrologist with a community pediatric practice following complex patients such as childhood cancer survivors. R.P has expertise in providing complex care in the community. |
| Peter Wong | P.W. is a general pediatrician and cardiologist with a community pediatric practice following complex patients such as childhood cancer survivors. P.W. has expertise in providing complex care in the community and in performing primary practice research. |
| Laura Wheaton | L.W. is paediatric hematologist oncologist at Queen’s University. She is a new investigator developing expertise in health education and adolescent oncology. L.W. is a co-site investigator in Kingston. |
| Paul Gibson | P.G. is the POGO chief clinical officer, and pediatric hematologist-oncologist at Western University. He is a key policy maker, stakeholder, and liaison for POGO (leads provincial clinic development) and other provincial and national cancer societies. |
| Kirby Kim | Patient Partner: K.W. is a parent of a childhood cancer survivor who experienced kidney disease or hypertension due to therapy. K.W. has contributed to current proposal ideas and will review results early and periodically and will provide expertise for knowledge translation. K.W is proficient in communicating with other families and performing computer skills. |
| **Trainee** | |
| Asaf Lebel | A.L. was a post-doctoral research fellow at Sickkids, currently a Pediatric Nephrologist in Israel. He contributed by elaborating on Aim 2 and completing other funded work that will complement work from these studies. A.L. will assist in interpreting ambulatory blood pressure monitoring and scientific input. |

Abbreviations: POGO=Pediatric Oncology Group of Ontario

**Supplementary Table 2.** 24-hour ambulatory blood pressure monitoring (ABPM) parameters for ABPM abnormalities and ABPM-hypertension

|  | **Casual BP** | Mean ABPM  Systolic BP ±Diastolic BP  (day, night, or 24hr) | Systolic BP ±Diastolic BP  load (% abnormal BP’s)  (day, night or 24hr) |
| --- | --- | --- | --- |
| **White coat hypertension** | Office hypertension | < 95^th^ %tile | < 25 % |
| **Masked hypertension** | No Office hypertension | ≥ 95^th^ %tile | ≥ 25 % |
| **Pre-hypertension** | Office hypertension | <95^th^ %tile | ≥ 25 % |
| **Ambulatory hypertension** | Office hypertension | ≥ 95^th^ %tile | 25-50 % |
| **Severe hypertension** | Office hypertension | ^th^%tile | > 50 % |
| **Non-BP dipping** | ([mean day BP]–[mean night BP])/mean day BPx100 <10% | | |

Abbreviations: BP=Blood pressure; ABPM=24-hour ambulatory blood pressure monitoring; hr=hour

Note:

To conduct ABPM, a patient wears an inflatable cuff for 24-hours (day and night). The cuff inflates every 20-30 minutes. Office hypertension is defined in Table 5 based on age-, height- and sex- percentiles. Casual BP is a BP measure at a single visit. BP load is the percentage of abnormally elevated BP readings for age-, sex-, and height- percentiles. Non-BP dipping is a lack of normal BP dip during sleep, more precisely defined as a decrease of less than 10% in the average nighttime SBP or DBP compared to the average daytime SBP or DBP measure. Patients with casual BP > 95^th^ percentile, mean ambulatory SBP or DBP > 95^th^ percentile and ≥ 25% load are determined to have ambulatory hypertension; if the patient met the above criteria for ambulatory hypertension but had a non-hypertensive casual BP (<95^th^ percentile), they were determined to have masked hypertension (a form of ambulatory hypertension). All percentiles listed are age, sex and height adjusted.

**Supplementary Information References**

1. Pole JD, Gu LY, Kirsh V, et al. Subsequent Malignant Neoplasms in a Population-Based Cohort of Pediatric Cancer Patients: A Focus on the First 5 Years. *Cancer Epidemiol Biomarkers Prev* 2015; 24: 1585-1592. 20150719. DOI: 10.1158/1055-9965.Epi-15-0360.

2. Nathan PC, Nachman A, Sutradhar R, et al. Adverse mental health outcomes in a population-based cohort of survivors of childhood cancer. *Cancer* 2018; 124: 2045-2057. 20180222. DOI: 10.1002/cncr.31279.

3. Nathan PC, Bremner KE, Liu N, et al. Resource Utilization and Costs in Adolescents Treated for Cancer in Pediatric vs Adult Institutions. *J Natl Cancer Inst* 2019; 111: 322-330. DOI: 10.1093/jnci/djy119.

4. Chellapandian D, Pole JD, Nathan PC, et al. Congestive heart failure among children with acute leukemia: a population-based matched cohort study. *Leuk Lymphoma* 2019; 60: 385-394. 20180703. DOI: 10.1080/10428194.2018.1474522.

5. Greenberg ML, Barr RD, DiMonte B, et al. Childhood cancer registries in Ontario, Canada: lessons learned from a comparison of two registries. *Int J Cancer* 2003; 105: 88-91. DOI: 10.1002/ijc.11004.
